# CFD Simulation of Spread Risks of Infectious Disease due to Interactive Wind and Ventilation Airflows via Window Openings in High‐Rise Buildings

**DOI:** 10.1063/1.3452160

**Published:** 2010-05-21

**Authors:** J. L. Niu, N. P. Gao

**Affiliations:** a Department of Building Services Engineering, The Hong Kong Polytechnic University; b School of Mechanical Engineering, Tongji University; City University of Hong Kong; City University of Hong Kong; University of Macau (UM); University of Macau (UM)

## Abstract

One of the concerns is that there may exist multiple infectious disease transmission routes across households in high‐rise residential buildings, one of which is the natural ventilative airflow through open windows between flats, caused by buoyancy effects. This study presents the modeling of this cascade effect using computational fluid dynamics (CFD) technique. It is found that the presence of the pollutants generated in the lower floor is generally lower in the immediate upper floor by two orders of magnitude, but the risk of infection calculated by the Wells‐Riley equation is only around one order of magnitude lower. It is found that, with single‐side open‐window conditions, wind blowing perpendicularly to the building may either reinforce or suppress the upward transport, depending on the wind speed. High‐speed winds can restrain the convective transfer of heat and mass between flats, functioning like an air curtain. Despite the complexities of the air flow involved, it is clear that this transmission route should be taken into account in infection control.

### INTRODUCTION

Airborne transmission is known to be the route of infection for a number of diseases including smallpox, tuberculosis, and severe acute respiratory syndrome (SARS). With the lessons from SARS outbreak in 2003 and the threats from other influenza pandemic, our concern on aerosol-transmitted infections in hospitals buildings, residential buildings, and even in transportation vehicles has been refreshed. Li et al. (2006) reviewed over 40 studies on the relationship between the transmission of infection and ventilation systems and found that there was sufficient evidence to demonstrate the association between ventilation and indoor air movements and the transmission and spread of infection diseases. Anecdotal evidences in Hong Kong during the SARS outbreak in the spring of 2003 are that, in certain high-rise residential buildings, and SARS virus was found within the deposits on the window-sill and floors, immediately beneath which, residents of the floors had been infected with SARS. These blocks have rectangular plan layouts and with common-corridors separating the two sides, each of which has a flat-façade with operable windows. With doors closed, the flats become single-sided natural ventilated, and the open windows function as both inlet and outlet of air. Room exhaust air, which is typically not centralized and not stacked, is left to drift freely around the building. Therefore the exhaust air of one flat may become the intake of the adjacent upper flat. Under normal circumstances, one can detect from the smell of the air what one's neighboring is cooking. This fact arouses our concern on the vertically upward transport of contaminants between flats in high-rise residential buildings with natural ventilation, i.e., there may exist a cascade effect, which is over-looked so far in both building design and infection control.

In recent years, natural ventilation has attracted considerable interest in the designs of green buildings (Allard 1998). There are two major forms of natural ventilation: single-sided and cross flow. Cross ventilation generally promotes a robust airflow through an internal space via multiple openings on different facades. However, in densely-

CP1233 *The 2nd International ISCM Symposium and The 12th International EPMESC Conference*
J. W. Z. Lu, A.Y.T. Leung, V. P. Iu, and K. M. Mok
© 2010 American Institute of Physics 978-0-7354-0778-7/10/$30.00

occupied urban environments, there may be only one external façade for small cellular rooms. The design of buildings thus often adopts single-sided natural ventilation. In this configuration, wind turbulence and temperature difference between indoor and outdoor are the main driving forces. Temperature-driven single-sided natural ventilation through large openings has been well studied in regard to air change rate (Favarolo and Manz 2005) and the resulting indoor air quality and comfort level (Gan 2000) by experimental works (Heiselberg et al. 2001), theoretical predictions (Fracastoro et al. 2002), and CFD simulations (Jiang and Chen 2003, Niu et al 1992, Allocca et al. 2003). However less consideration has been given to the fate of the exhaust air, on whether it will re-enter the upper floors.

From the perspective of infection control, this cascade air flow is undesirable. Earlier special onsite investigation after the SARS outbreak revealed the room immediately upstairs could contain up to 7% of the exhaust air from the lower floor (Niu and Tung 2007). Therefore current study applies the computational fluid dynamics (CFD) method to investigate the cascade effect between flats in high-rise residential buildings with pure buoyancy-driven single-sided natural ventilation through a large rectangular opening. Further, the combined effect of wind and buoyancy on the contaminant transport upward is inspected. The investigation is focused on the passive tracer gas concentration field. Some of the results are compared with the earlier on-site measurements (Niu and Tung 2008). The aerosols' movement modeling, which can more really capture the dispersion characteristics of sneezed/coughed virus-containing droplets, will be studied and reported in a separate paper(Gao et al 2009).

### CFD METHODS VALIDATION

The turbulent effect is simulated by the re-normalization group (RNG) k-ε model. All thermophysical properties are assumed to be constant except for density, which is treated with the Boussinesq approximation. The convection terms are discretized by second order upwind scheme and the diffusion term by centraldifference also with second-order accuracy. The SIMPLE algorithm (Partankar 1980) is adopted as pressure-velocity coupling method. The elementary flow characteristics involved in present study are natural convection flow in a cavity and forced convection flow over a bluff body. As validations of the above model, the simulations on natural convection and forced convection are compared with the measured data from the literature(Gao et al 2008). These flows include a two-dimensional buoyancy flow in an enclosed cavity, experimentally investigated by Cheesewright et al. (1986), the airflow around a building-like model (Jiang et al. 2003) and Pure buoyancy-driven single-sided natural ventilation through a large opening (Heiselberg et al. 2003).

### CONFIGURATION OF SIMULATION

To study the possible cross contamination of ventilation air via open windows in multi-family buildings, a four-storey building is adopted (Figure 1). Windows are opened at the windward at the second and third floor. The room dimension is height(Y) × length(X) × width(Z) = 2.7m × 3.1m × 2.4m and the window height(Y) × width(Z) = 1.2m × 0.75m. The bottom of the window is 0.8m above the room floor. These dimensions are identical with those in our field study. This building is placed in a computational domain, as illustrated in Figure 10. According to the experiences by Schaelin et al. (1992) and Allocca et al. (2003), this domain is large enough to obtain the true results. The domain boundary at x-y plane is defined as symmetry. It means the building and its surroundings are extended in the negative and positive z direction. This treatment is based on the fact that in many high residential blocks in Hong Kong the shape of the whole building is like a vertical slab.

Heat is released only from the internal walls at the second and third floors. The atmospheric air temperature is 20 ℃ and the indoor wall surface temperature is 25℃. In the simulation of a low wind day, a uniform velocity profile (0.1m/s) is set at the domain inlet, in place of pressure boundary because we find it is difficult to reach convergence if using pressure boundary at the domain inlet as well as outlet. In a normal urban environment, even in a windless day in meteorology, the wind speeds are usually higher than this value (0.2m/s). Therefore a small meteorological wind speed, Vmet(wind speed usually taken at 10m above the ground level), at 0.5m/s, 1.0m/s, 2.0m/s, and 4.0m/s is considered. The wind profile in an urban environment is calculated by

$$V_y = 0.35 V_{met} y^{0.25} \quad (1)$$

The turbulence on the inlet boundary is characterized by turbulence intensity and length scale, which are 8% and 1m, respectively. Carbon dioxide ($CO_2$), as a tracer of indoor pollutants, is generated at a rate of 8mg/s in the middle of the second floor at the height of 1.6m. The governing equation of this tracer gas is solved alone after the convergence of air flow field.

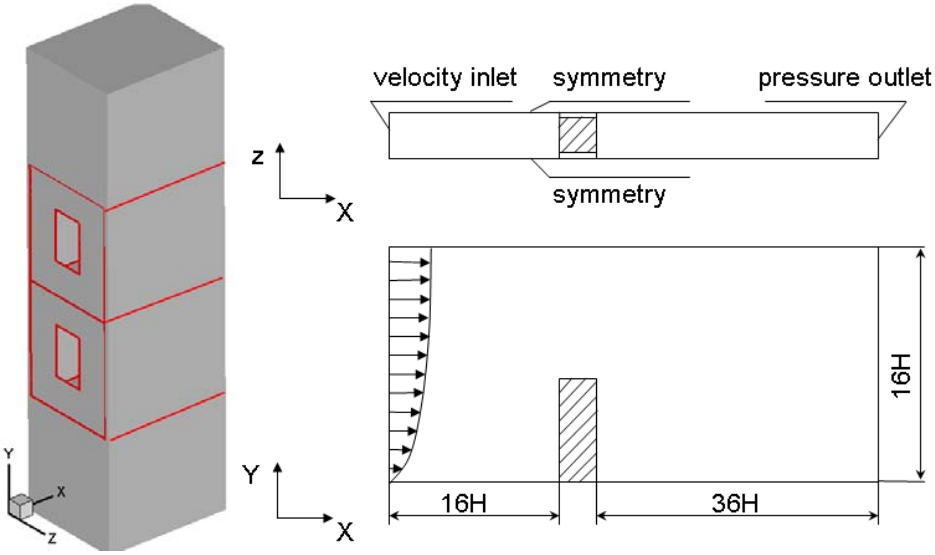

**FIGURE 1.** Description of the building model and the computational domain

### RESULTS AND DISCUSSIONS

#### *Air Change Rate (ACH)*

Tracer gas decaying as a function of time is plotted in Figure 2. At time = 0 s, the room is uniformly filled with tracer gas. Then the air exchange through the window is permitted and the normalized volume-average concentration is monitored. As seen from Figure 2, a gentle wind up to 1.0 m/s normal to the window has no or little influence on the ACH. However if the normal wind speed increases to 2.0 and 4.0 m/s, the ACH is remarkably lowered. This finding is supported by the experiment by Wilson and Kiel (1990). The effects of weak wind are multiple and complicated. Firstly, wind increases the intensity of outdoor turbulence, and further increases the interfacial mixing at the middle height of the opening between incoming and outgoing airstreams (Figure 12). This mixing lowers the net flow rate. It is the re-entrainment by interfacial mixing that produces the gradual profile of velocity and temperature along the height of the opening. Secondly, approaching wind speed profile may attenuate the net pressure difference across the opening (Figure 3). Wind and stack forces counteract each other. Thirdly, turbulent fluctuations in wind contain high frequency energy, which could strengthen the air exchange through the opening (Allard 1998). The frequency of turbulence energy for wind-driven flow is one order of magnitude higher than that for buoyancy-driven flow (Jiang and Chen 2001; 2003). In buoyancy-driven cases mean pressure difference across the opening impels airflow while on the other hand pressure fluctuation plays an important role in wind-driven natural ventilation. In Figure 2, the ACH is higher in the second floor than in the third floor at a wind speed of 1.0, 2.0, and 4.0 m/s. This discrepancy was observed in our field study as well.

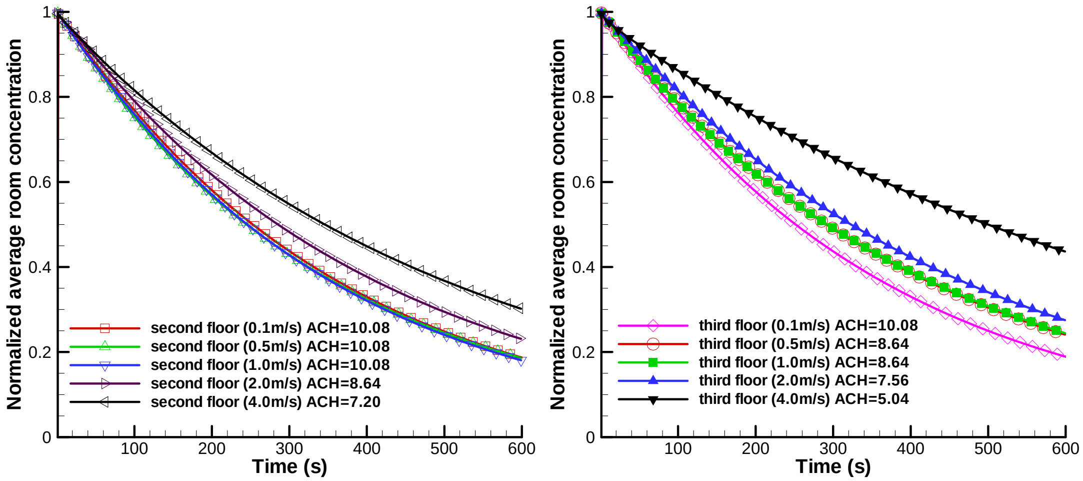

**FIGURE 2.** Decaying curves of normalized average indoor concentration

#### *Cascade Effect on Infection Spread*

Mass fractions of the tracer gas at the middle section in z direction are shown in Figure 3. In the windless condition, the warm plume emitted from the upper part of the lower window drifts upward. Due to the horizontal momentum when leaving the lower window, the central region of this plume is apart from the upper window. The average concentration in the upper room is two orders of magnitude lower than in the lower room. As wind speed ascends from 0.5 to 2.0m/s, the plume is forced to approach the upper window by the impingement of the normal wind. The re-entry ratio increases to up to 16.3% as a result. The wind assists in the cascade effect. If the wind speed increase further to 4.0 m/s, the development of warm plume from the lower window is confined. The air velocity levels in the two rooms (mean value 0.08 m/s) are quite lower than in windless case, which results in a diffusion-dominated spread of pollutants. The re-entry ratio is the lowest 3.5%. It seems that strong normal wind will act as an air curtain. This curtain helps to block the heat and mass transfer between flats.

Based on the knowledge of infection dose (the number of organisms required to cause infection), the risk of airborne infection and ventilation rate can be correlated by Wells-Riley equation (Riley et al. 1978):

$$P = C/S = (1 - e^{-Iqpt/Q}) \tag{6}$$

The quantum, $q$, represents the generation rate of infectious doses. The Wells-Riley equation is set up on the assumption of well-mixing and steady-state condition. Here using CFD we can get the concentration of infectious particles at a certain point, which allows derivation of spatial distribution of infection risk. It does not require the assumption of well-mixing conditions. The spatial variance of infection probability is similar with the distribution of mass fraction of the tracer gas (Figure 14). Supposing one patient standing at the middle of the second floor producing 13 infectious quanta per hour ($q = 13$), pulmonary ventilation rate to be 0.6 m3/h ($p = 0.6$), and exposure time of 8 hours ($t = 8$), the calculated mean infection probabilities are listed in Table 2. A probability as high as 6.6 % in the third floor alerts us that the upward transport of infectious diseases in high-rise residential buildings is worth of due considerations in infection control. As one of the effective intervention measures is to isolate and quarantine the close-contacts, the upstairs household residents may be included in the close-contact list in case of a highly infectious emerging disease.

**TABLE 1.** Mean risk of infection from Wells-Riley equation and re-entry ratio in various cases

| | 0.1 m/s | 0.5 m/s | 1.0 m/s | 2.0 m/s | 4.0m/s |
|---|---|---|---|---|---|
| Mean risk of infection (second floor) | 30% | 28% | 29% | 31% | 46% |
| Mean risk of infection (third floor) | 2.0% | 3.4% | 3.5% | 6.6% | 1.7% |
| Re-entry ratio | 7.5% | 9.6% | 10.9% | 16.3% | 3.5% |

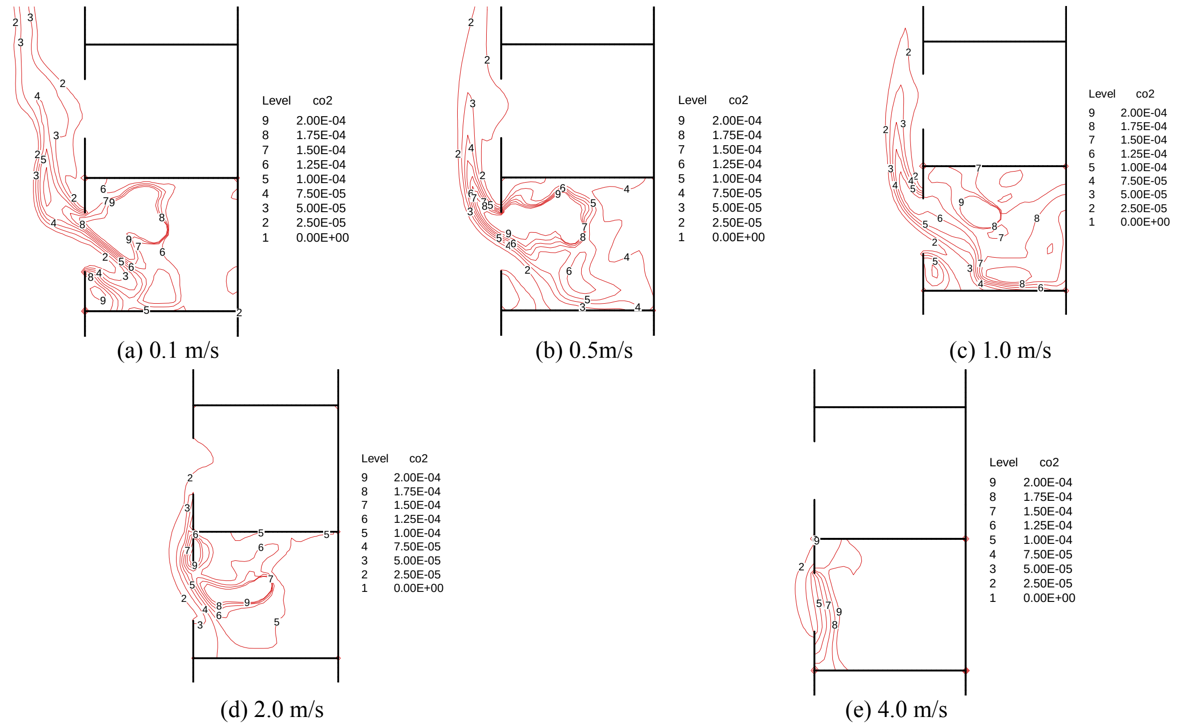

**FIGURE 3.** Distribution of mass fraction (kg/kg) of tracer gas $CO_2$ which is generated in the middle of the second floor at a rate of 8 mg/s

## CONCLUSIONS

It is found that, in a windless day, around 7.5% of the exhaust air can be re-entrained into the upper room. The concentration level is generally two orders of magnitude lower in the adjacent upper room than in the lower source room, but the risk of infection is only one order of magnitude lower and is still significantly high when it was assessed using the Wells-Riley TB infection model. One of the parameters is the long time residents would spend at their own homes. The effect of wind blowing perpendicularly to the window is rather complicated. It may reinforce or prevent the upward transport, depending on the wind speed. A gentle-wind forces the warm polluted plume to enter into the upper window by its horizontal momentum. But high-speed winds may function like an air curtain, suppressing the convective spread of pollutants between flats. In spite of these complexities, the vertical spread risks should not be overlooked from the perspective of infection control, especially when an emerging infectious disease is dealt with. Present simulations using the RNG k-ε model are not able to reveal the turbulent fluctuations and instantaneous air exchanges through the opening, especially in wind-driven single-sided natural ventilation, although we find the value of ACH from the RNG k-ε model is acceptable. The symmetrical boundary conditions imposed to the pollutant concentration equations in the horizontal directions may have overestimated the vertical transmission, but the effects are expected to be small, and will be examined in our further studies.
